# Predictive Factors for Long-Term Disease Control in Systemic Treatment-Naïve Oligorecurrent Renal Cell Carcinoma Treated with Up-Front Stereotactic Ablative Radiotherapy (SABR)

**DOI:** 10.3390/cancers16172963

**Published:** 2024-08-25

**Authors:** Ciro Franzese, Veronica Vernier, Marco Badalamenti, Raffaella Lucchini, Sara Stefanini, Anna Bertolini, Maryia Ilieva, Luciana Di Cristina, Beatrice Marini, Davide Franceschini, Tiziana Comito, Ruggero Spoto, Luca Dominici, Carmela Galdieri, Pietro Mancosu, Stefano Tomatis, Marta Scorsetti

**Affiliations:** 1 Department of Radiotherapy and Radiosurgery, IRCCS Humanitas Research Hospital, Via Manzoni 56, Rozzano, 20089 Milan, Italy; 2 Department of Biomedical Sciences, Humanitas University, Via Rita Levi Montalcini 4, Pieve Emanuele, 20072 Milan, Italy

**Keywords:** renal cell carcinoma, oligorecurrence, SABR, radiotherapy, predictive factors

## Abstract

**Simple Summary:**

Metastatic renal cell carcinoma (RCC) represents a significant challenge in oncology, and systemic therapy remains the primary treatment modality. Radiation therapy (RT) offers a non-invasive ablative approach to the management of oligometastatic disease, and the use of Stereotactic Ablative Radiotherapy (SABR) has demonstrated remarkably high disease control. Within the broad spectrum of oligometastatic disease, we evaluated the efficacy of SABR on oligorecurrent RCC systemic treatment-naïve, and we aimed to identify predictive factors for long-term disease control. We demonstrated the potential of SABR to delay the need for systemic therapy and confirmed the long-term efficacy of SABR for oligometastatic RCC patients, with a superior benefit for patients with single lung metastasis, potentially refining patient selection processes to tailor treatments.

**Abstract:**

Background: Stereotactic ablative radiotherapy (SABR) is emerging as a potential local treatment option for oligometastatic RCC. This study aims to evaluate the efficacy of SABR in patients with oligorecurrent RCC. Methods: A total of 50 patients with histologically confirmed RCC underwent SABR for oligorecurrence between 2006 and 2022. Eligible patients had up to five extracranial metastases and were systemic treatment-naïve at the time of irradiation. The primary endpoints of the analysis were overall survival (OS), local control (LC), distant metastasis–free survival (DMFS), and time to systemic therapy initiation. Results: The median OS was not reached, with 1- and 3-year OS rates of 93.8% and 77.5%, respectively. LC rates at one and three years were 95.8% and 86.5%, respectively. The median time to systemic therapy initiation was 63.8 months, and the median DMFS was 17.9 months, with one- and three-year rates of 63.4% and 36.6%, respectively. Multiple metastases were a negative predictive factor for DMFS (HR 2.39, *p* = 0.023), whereas lung metastases were associated with a more favorable outcome (HR 0.38, *p* = 0.011). Conclusions: SABR offers a valuable treatment option for oligometastatic RCC, demonstrating significant potential for achieving long-term disease control and delaying the need for systemic therapy.

## 1. Introduction

Renal cell carcinoma (RCC) accounts for approximately 3 to 5% of all adult malignancies [1]. At the time of diagnosis, about 15% of patients present with metastatic disease, with an additional 15 to 25% developing distant metastasis after treatment of the primary tumor [2], which is generally associated with a poor prognosis and a 5-year survival rate of about 15% [3,4]. A specific subset of metastatic patients, termed oligometastatic, is characterized by a limited metastatic burden, with one to five distant metastases [5]. Despite systemic therapy being the standard of care for these patients, the potential of metastasis-directed therapy (MDT) to enhance disease control, patients’ quality of life (QoL), and survival has been explored, showing promising results across various primary tumors [6].

Compared to more invasive approaches, radiation therapy (RT) offers a non-invasive alternative for the management of oligometastatic disease with well-established efficacy and safety profiles. Historically, RCC has been categorized as a radioresistant tumor, and RT was used exclusively for symptom palliation [7]. However, the introduction of Stereotactic Ablative Radiotherapy (SABR) has shifted this perspective: delivering higher doses per fraction with an ablative effect results in a strong dose effect for RCC, encompassing the well-known radioresistance associated with this histology. The use of SABR in oligometastatic RCC has demonstrated remarkably higher disease control compared to conventional radiotherapy (RT) [8], with 1-year local control (LC) rates reaching up to 90% and showing favorable toxicity profiles [9,10,11].

Within the broad spectrum of oligometastatic presentations, oligometastatic diseases can be further subclassified into oligorecurrence, oligoprogression, and oligopersistence, considering whether the oligometastatic disease is diagnosed during a treatment-free interval or during active systemic therapy and whether or not an oligometastatic lesion is progressing on current imaging [12]. The diagnoses of metachronous oligorecurrence have generally been associated with better outcomes compared to synchronous oligometastatic disease and oligoprogressive settings. Consequently, the use of up-front MDT, such as SABR, in selected oligorecurrent patients could potentially secure long-term disease control, postponing the need for systemic therapy. Ablative metastasis-directed radiotherapy may play a key role in the clinical management of these selected patients by offering a non-invasive treatment delivered in 3 to 6 daily fractions with promising results in terms of efficacy and toxicity profile, minimizing the impact of the beginning of polypharmacologic systemic therapy on QoL.

Despite the fact that the role of SABR in the patients affected by oligometastatic RCC patients is currently under investigation, a harmonious consensus on the treatment management of this complex entity is still lacking. Therefore, the aim of the present study was to evaluate the role of SABR in the specific setting of oligorecurrent RCC patients who have not received systemic treatment yet and to identify predictive factors for long-term disease control in order to provide new insights on unmet clinical needs in this scenario.

## 2. Materials and Methods

This single-center retrospective study analyzed patients with histologically confirmed RCC who had a diagnosis of oligorecurrence treated with SABR between 2006 and 2022. Eligible patients included those with up to five extracranial metastases, all treated with SABR, and who were systemic treatment-naïve at the time of irradiation. The multidisciplinary uro-oncology team approved all treatments. Written informed consent was obtained from all patients, and the study received approval from the local ethics committee (N. 22/20).

Patients underwent simulation with a CT scan with 3 mm slice thickness in a supine position, immobilized with a thermoplastic mask, and customized according to the lesion location. The motion of lesions in the lungs or upper abdomen was assessed using 4D CT scans. Contrast enhancement was employed according to clinical practice standards to improve disease site visualization. All patients were treated with Volumetric Modulated Arc Therapy (VMAT) and were evaluated daily with cone-beam computed tomography prior to each treatment session. The dose and treatment schedule were determined based on the site of the disease and the proximity of organs at risk. After treatment, patients received clinical and radiological follow-ups every 3 months, with tumor response and toxicity patterns documented at each visit. Acute and late adverse events were classified using the Common Terminology Criteria for Adverse Events Version 5.0.

The endpoints of the study were overall survival (OS), LC, distant metastasis–free survival (DMFS), and time to systemic therapy initiation. Kaplan–Meier analysis was used to calculate survival outcomes, and the log-rank test was performed to assess survival distributions across different patient subgroups. To estimate the effect of potential risk factors on survival outcomes, univariate Cox proportional hazards regression was used, providing hazard ratios (HRs) with confidence intervals (CIs) for each variable. A multivariable stepwise Cox regression analysis was conducted to identify the most significant clinical factors associated with survival outcomes, using a significance threshold of *p* < 0.05. Statistical calculations were performed using STATA, version 18.

## 3. Results

Our analysis included 50 patients with a total of 81 oligometastatic lesions from RCC treated with SABR. Patients’ characteristics are summarized in Table 1. The cohort predominantly consisted of males (42 patients, 84.0%) with an ECOG performance status (PS) of 0 (33 patients, 66.0%). The median age at the time of SABR was 66 years (range 44–84). SABR was administered to a single metastasis in 29 patients (58.0%), to two metastases in 15 patients (30.0%), and to three to five metastases in 2 patients (12.0%). Forty-one patients (82.0%) had a lesion in one organ, eight patients (16.0%) had lesions across two organs, and one patient (2.0%) had lesions in three organs. The median biologically effective dose (BED) was 78.7 Gy (range 35.7–120 Gy), calculated using an α/β ratio of 10.

**Figure 1:**
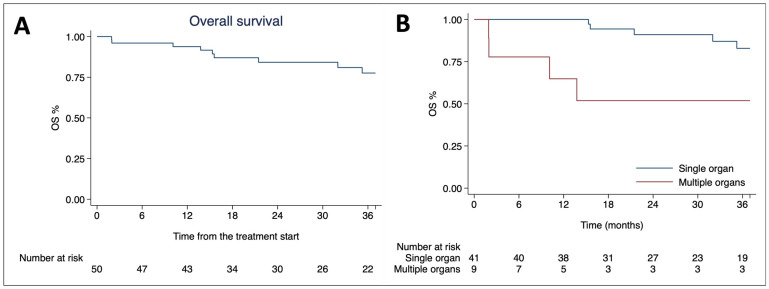
Kaplan–Meier curves of overall survival for the entire population (**A**) and stratified according to the number of treated organs (**B**).

The median follow-up period was 33.2 months (range 6–213). The median OS was not reached, with one- and three-year OS rates of 93.8% (95% CI 82.2–97.9) and 77.5% (95% CI 60.6–87.9), respectively, as depicted in Figure 1A.

Univariate analysis revealed that both the presence of single versus multiple metastases (HR 4.93, 95% CI 1.33–18.28; *p* = 0.017) and the number of treated organs (HR 5.30, 95% CI 1.90–14.81; *p* = 0.001) significantly correlated with OS. However, the multivariable analysis identified only the number of treated organs as an independent negative predictive factor for OS (HR 3.59, 95% CI 0.99–12.98; *p* = 0.050), as shown in Table 2. The median OS for patients treated on a single organ was not reached, compared to 59.0 months for those treated at multiple organ sites (*p* = 0.003), as shown in Figure 1B.

Median LC was not reached, with one- and three-year LC rates of 95.8% (95% CI 84.3–98.9) and 86.5% (95% CI 69.7–94.3), respectively. No factors were statistically significant as independent predictors of improved LC in both univariate and multivariable analyses (Table 3).

Thirty-three patients (66.0%) had a progression of disease with the appearance of new metastases. Fifteen patients (30%) underwent further SABR for repeated oligometastatic disease. The median time to systemic therapy initiation was 63.8 months, with 1- and 3-year rates of 93.7% (95% CI 81.7–97.4) and 59.7% (95% CI 40.7–74.5).

The median DMFS was 17.9 months, with one- and three-year rates of 63.4% (95% CI 47.9–75.4) and 36.6% (95% CI 22.3–51.0), respectively. Multivariate analysis revealed that having multiple metastases was a negative predictive factor for DMFS (HR 2.39, 95% CI 1.12–5.08; *p* = 0.023), whereas lung metastases were associated with a favorable outcome (HR 0.38, 95% CI 0.18–0.80; *p* = 0.011), as illustrated in Table 4.

**Figure 2:**
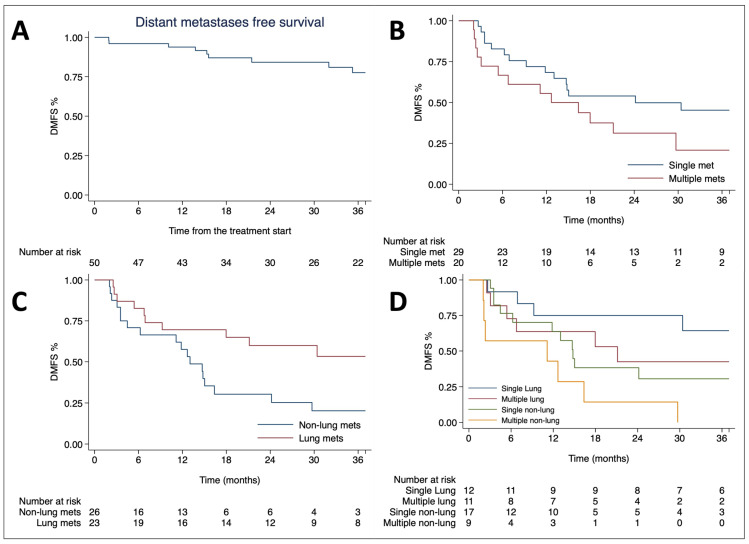
Kaplan–Meier curves of distant metastases free survival for the entire population (**A**), stratified by the number of metastases (**B**), the site of metastases (**C**), and combined risk factors (**D**).

The median DMFS for patients with single metastases was 24.1 months compared to 12.6 months for those with multiple metastases (*p* = 0.098) and was 42.0 months for lung metastases versus 13.0 months for non-lung metastases (*p* = 0.016). When stratifying patients by both risk factors, the median DMFS was 42.1 months for those with a single lung lesion, 21.1 months for those with multiple lung lesions, 14.8 months for a single non-lung lesion, and 11.1 months for multiple non-lung metastases (Figure 2).

## 4. Discussion

The outcomes of our study highlight the potential of SABR as a pivotal treatment modality for patients with oligorecurrent RCC. While systemic therapy remains the cornerstone for stage IV metastatic RCC, it achieves complete eradication of gross metastatic lesions in only about 10% of patients [13]. Our finding demonstrated long-term disease control after SABR, with 86.5% of patients free from in-field progression three years post-treatment. This compares favorably to a meta-analysis conducted by Zaorsky et al. [14], which reported 89.1% of 1-year LC across 28 studies for extracranial metastases treated with SABR.

The primary objectives of SABR in the context of metastatic RCC may include different purposes, from extending the duration of ongoing systemic treatment by ablating isolated foci of resistance in the setting of oligoprogressive disease to deferring the initiation of systemic therapy in oligorecurrent patients. Given the favorable toxicity profile associated with SABR [15], it stands out as a non-invasive alternative to more aggressive treatments, which is pivotal considering the substantial impact of treatment-related adverse effects on patients’ QoL. With limited evidence currently supporting the benefit of SABR in systemic treatment-naïve oligorecurrent RCC patients, our study aimed to assess the efficacy of SABR in delaying the initiation of systemic therapy in oligometastatic RCC patients. We demonstrated a median time to the initiation of systemic therapy of 63.8 months, with more than half of the patients remaining free from systemic treatment three years after SABR.

The feasibility of deferring systemic therapy onset in carefully selected oligometastatic RCC patients has been investigated by a few prospective and retrospective studies. Two phase II trials specifically examined the implementation of SABR as an alternative to systemic therapy for oligorecurrent RCC. Tang et al. [16] treated with definitive intent RT a total of 30 metastatic RCCs with fewer than six lesions. At a median follow-up of 17.5 months, the authors demonstrated 1-year PFS of 64% and systemic therapy–free survival of 82%, with two grade 3 and one grade 4 adverse events. Similarly, Hannan et al. [17] described the outcome of 23 RCC patients with 57 metastases treated with SABR. The study met its primary endpoint with 1-year freedom from systemic therapy in 91.3% of enrolled patients. The treatment was well tolerated, and when compared with the baseline, there was no significant decline in QoL.

The largest retrospective analysis was conducted by Zhang et al. [18], including 47 patients treated with SABR on 88 metastases. They reported a median freedom from systemic therapy from the first SABR of 15.2 months. An improved outcome was observed in patients with metachronous disease (HR 2.67; *p* = 0.02), solitary metastasis (HR 2.26; *p* = 0.05), and non-bone metastasis (HR 2.21; *p* = 0.04).

Identifying patients who will achieve long-term disease control with SABR without the early emergence of new metastases remains an area of ongoing research. In our analysis, the differentiation between patients with single versus multiple metastases and the location of the lesions in the lung versus other organs provide new insights into the influence of disease characteristics on the SABR’s objectives. We observed the least favorable DMFS in patients with multiple non-lung oligometastases (median 11.1 months), whereas the best outcome was seen in patients treated for single lung lesions (median 42.1 months).

Additional predictive factors were reported in the published literature. Singh et al. [19] identified in 115 metastatic RCC patients with 181 lesions treated with SABR that both Karnofsky Performance Score (KPS) and osseous metastatic disease were predictors of OS. In another cohort of 207 oligometastatic RCC, of whom 42.9% had oligorecurrent disease, the increasing number of metastases (HR 1.40; *p* = 0.003) together with the presence of bone disease (HR 2.62; *p* = 0.038) were independent predictors of progression-free survival (PFS) [20]. Liu et al. [21] investigated the impact of the extent of SBRT ablation in 101 RCC patients with up to 5 lesions. The complete ablation group had significantly longer PFS (26.0 versus 18.8 months; *p* = 0.043) and cancer-specific survival (not reached versus 55.3 months; *p* = 0.012) compared with the no or incomplete SABR group, especially in patients with age < 55 years, ECOG PS of 0–1, clear-cell histology, IMDC intermediate/poor risk, metachronous metastasis, and fewer than three lesions.

Despite the valuable insights provided, our study has limitations, including its retrospective nature and single-center design, which may introduce bias and restrict the generalizability of our findings. The small sample size and heterogeneity of the patient population concerning disease sites warrant a careful interpretation of the results; in addition, patients treated with brain metastases were excluded. Moreover, the lack of data on prognostic models from the Memorial Sloan Kettering Cancer Center (MSKCC) and the International Metastatic Renal Cell Carcinoma Database Consortium (IMDC) presents another layer of bias in our study. We acknowledge that the partial overlap of patients with a previous study may introduce some limitations in terms of bias and generalizability, and we have taken this into consideration by conducting sensitivity analyses to ensure the robustness of our findings. Future prospective studies with larger cohorts are needed to validate our results; taking into account patients’ characteristics, disease features, and prognostic factors could help patients’ selection. Additionally, identifying novel predictive biomarkers of treatment response could enhance treatment personalization.

## 5. Conclusions

Our study contributes new insights to the expanding evidence base for the application of SABR in managing oligometastatic RCC. We confirmed the long-term efficacy of SABR for oligorecurrent RCC patients, with a superior benefit for patients with single lung metastases over those with multiple or non-lung metastases. This distinction underscores the potential for refining RCC patient selection processes to tailor treatments more effectively. We demonstrated that SABR might play a key role in the clinical management of patients affected by oligorecurrence disease by delaying the onset of systemic therapy and offering a well-tolerated local treatment. Future prospective studies with larger cohorts are needed to validate these findings and further refine the criteria for selecting patients most likely to benefit from SABR. Moreover, identifying biomarkers predictive of treatment response could enhance patient selection and treatment personalization.

## Figures and Tables

**Table 1:** Patients’ and treatment’s characteristics.

**Treated Patients**	50
**Treated Oligometastases**	81
**Age**, median (range)	66 years (44–84)
**Gender**	
Female	8 (16.0%)
Male	42 (84.0%)
**ECOG Performance status**	
0	33 (66.0%)
1	15 (30.0%)
2	2 (4.0%)
**Number treated metastases**	
1	29 (58.0%)
2	15 (30.0%)
3	3 (6.0%)
4	2 (4.0%)
5	1 (2.0%)
**Treated organs**	
1	41 (82.0%)
2	8 (16.0%)
3	1 (2.0%)
**Site of treatment**	
Bone	4 (8%)
Liver	2 (4%)
Lymph nodes	6 (12%)
Adrenal glands	7 (14%)
Pancreas	5 (10%)
Lung	20 (40%)
Lung and lymph nodes	3 (6%)
Lung and bone	1 (2%)
Lymph node and adrenal gland	1 (2%)
Lung and pancreas	1 (2%)
**Disease-free interval**, median (range)	34.0 months (0–266.6)
**BED10**, median (range)	78.7 Gy (37.5–120)

**Table 2:** Univariate and multivariable analyses for overall survival. In bold, risk factors with a *p*-value of ≤0.05.

	Univariate			Multivariable		
	HR	95% CI	*p*-Value	HR	95%CI	*p*-Value
**Age**	1.05	0.99–1.12	0.057	-	-	-
**Gender, Male**	1.01	0.22–4.66	0.984	-	-	-
**ECOG PS**	1.63	0.69–3.88	0.262	-	-	-
**DFI**	1.00	0.99–1.01	0.253	1.00	0.99–1.01	0.077
**Time to SBRT**	0.97	0.94–1.01	0.240	0.96	0.93–1.00	0.102
**Single vs. multiple mets**	**4.93**	**1.33–18.28**	**0.017**	3.31	0.69–15.84	0.133
**Number of organs**	**5.30**	**1.90–14.81**	**0.001**	**3.59**	**0.99–12.98**	**0.050**
**Lung mets**	0.61	0.19–1.94	0.406	-	-	-

**Table 3:** Univariate and multivariable analyses for local control.

	Univariate			Multivariable		
	HR	95% CI	*p*-Value	HR	95% CI	*p*-Value
**Age**	0.99	0.91–1.08	0.907	-	-	-
**Gender, Male**	-	-	-	-	-	-
**ECOG PS**	0.50	0.06–3.84	0.508	0.22	0.02–1.72	0.150
**DFI**	0.98	0.94–1.01	0.268	-	-	-
**Time to SBRT**	0.98	0.94–1.03	0.553	-	-	-
**Single vs. multiple mets**	1.19	0.19–7.15	0.848	-	-	-
**Number of organs**	1.83	0.20–16.47	0.588	5.55	0.40–76.59	0.200
**Lung mets**	0.23	0.02–2.07	0.192	0.90	0.00–1.17	0.066

**Table 4:** Univariate and multivariable analyses for distant metastasis–free survival. In bold, risk factors with a *p*-value of ≤0.05.

	Univariate			Multivariable		
	HR	95%CI	*p*-Value	HR	95%CI	*p*-Value
**Age**	1.00	0.96–1.03	0.993	-	-	-
**Gender, Male**	1.78	0.62–5.12	0.278	2.16	0.74–6.28	0.157
**ECOG PS**	1.15	0.63–2.09	0.636	-	-	-
**DFI**	0.99	0.99–1.00	0.872	-	-	-
**Time to SBRT**	0.99	0.97–1.00	0.242	-	-	-
**Single vs. multiple mets**	1.81	0.88–3.70	0.104	**2.39**	**1.12–5.08**	**0.023**
**Number of organs**	1.32	0.50–3.46	0.566	-	-	-
**Lung mets**	0.49	0.24–1.01	0.056	**0.38**	**0.18–0.80**	**0.011**

## Data Availability

Data will be available on the legal repository upon request.
